# SELF-PERCEPTION OF AGING AND ASSOCIATED CHARACTERISTICS AMONG OLDER ADULTS

**DOI:** 10.1093/geroni/igz038.3084

**Published:** 2019-11-08

**Authors:** Timothy L Barnes, Shirley Musich, Shaohung Wang, Sandra Kraemer, Charlotte Yeh

**Affiliations:** 1 Optum, Eden Prairie, Minnesota, United States; 2 UnitedHealthcare, Minneapolis, Minnesota, United States; 3 AARP Services, Inc., Washington, District of Columbia, United States

## Abstract

Positive self-perception of aging has been linked to better physical and psychosocial health outcomes among older adults. Negative self-perception of aging has been associated with poorer health consequences including depression, limited mobility, and mortality. Despite significant findings, the comprehensiveness and quality of self-perception of aging research still warrants further investigation, especially when identifying factors for intervention. Using a large random stratified sample of AARP Medicare Supplement insured members, age≥65 years, with continuous coverage for ≥12 months, self-perception of aging and various socio-demographic, medical, and psychosocial characteristics were examined using Chi-square and multivariate logistic regression models. Self-perception of aging was measured using the five-item Attitudes Towards Own Aging subscale. Characteristics of interest included age, gender, health status, resilience, purpose, optimism, social network, physical activity, depression, falls, vision, hearing, oral health, and sleep quality. Propensity weighting was used to adjust for potential survey non-response bias. Of weighted survey respondents (N=14,046), 59% exhibited a positive self-perception, while 41% exhibited a negative self-perception. Respondents with a positive self-perception were more likely to be healthier, younger (<75 years), more active (≥3days), less depressed, have more diverse social networks, higher resilience, and purpose. Negative self-perception was associated with poorer health, older age, depression, and poorer vision, hearing, oral health, and sleep quality. The strongest characteristics associated with positive self-perception were purpose, resilience, physical activity, and social networks. Depression and sleep quality were the strongest characteristics associated with negative self-perception. Interventions targeting these characteristics could be beneficial in promoting positive self-perception of aging and health over time.

